# Developing a risk score for undiagnosed prediabetes or type 2 diabetes among Saharawi refugees in Algeria

**DOI:** 10.1186/s12889-022-13007-0

**Published:** 2022-04-11

**Authors:** Sigrun Henjum, Victoria Telle Hjellset, Eivind Andersen, Merete Øyaland Flaaten, Marianne S. Morseth

**Affiliations:** 1 Faculty of Health Sciences, Oslo Metropolitan University, Oslo, Norway; 2 Faculty of Humanities, Sports and Educational Science, University of South-Eastern Norway, Horten, Norway

**Keywords:** Type 2 diabetes, Rrisk score, Area under curve, Refugees

## Abstract

**Aims:**

To prevent type 2 diabetes mellitus (T2D) and reduce the risk of complications, early identification of people at risk of developing T2D, preferably through simple diabetes risk scores, is essential. The aim of this study was to create a risk score for identifying subjects with undiagnosed prediabetes or T2D among Saharawi refugees in Algeria and compare the performance of this score to the Finnish diabetes risk score (FINDRISC).

**Methods:**

A cross-sectional survey was carried out in five Saharawi refugee camps in Algeria in 2014. A total of 180 women and 175 men were included. HbA1c and cut-offs proposed by the American Diabetes Association (ADA) were used to define cases. Variables to include in the risk score were determined by backwards elimination in logistic regression. Simplified scores were created based on beta coefficients from the multivariable model after internal validation with bootstrapping and shrinkage. The empirical cut-off value for the simplified score and FINDRISC was determined by Area Under the Receiver Operating Curve (AUROC) analysis.

**Results:**

Variables included in the final risk score were age, body mass index (BMI), and waist circumference. The area under the curve (AUC) (C.I) was 0.82 (0.76, 0.88). The sensitivity, specificity, and positive and negative predictive values were 89, 65, 28, and 97%, respectively. AUC and sensitivity were slightly higher and specificity somewhat lower than for FINDRISC.

**Conclusions:**

The risk score developed is a helpful tool to decide who should be screened for prediabetes or T2D by blood sample analysis. The performance of the risk score was adequate based on internal validation with bootstrap analyses, but should be confirmed in external validation studies.

## Background

The global age-standardized prevalence of type 2 diabetes (T2D) nearly doubled from 1980 to 2014 [1] causing great economic strain to health care systems [2] and increased risk of premature death globally [1]. In the Middle East and North Africa region (MENA), the prevalence of T2D was 12.8% in 2019 [3]. There has been a trend towards Westernized diets and a more sedentary lifestyle, with an expected doubling of diabetes prevalence in the next 20 years as a result [4]. At the same time, it is estimated that 45% of adults with T2D on a global scale are undiagnosed [5]. Tight control of blood glucose levels significantly reduces the risk of complications [6], and early diagnosis to prevent adverse health outcomes is essential [1].

Screening may identify individuals at high risk of developing diabetes so that appropriate preventive lifestyle measures can be implemented to reduce the risk of complications [2]. Meanwhile, lab-based screening of diabetes risk is costly, and the International Diabetes Federation (IDF) recommends performing blood tests only on high-risk individuals identified by screening questionnaires [7]. Screening forms that are easy to comprehend and do not include lab results may be suitable in low-resource settings [8]. Various diabetes risk assessment forms have been developed; some assess the risk of developing diabetes (incident cases) while others assess risk factors for having undiagnosed (prevalent cases) T2D [2]. The large majority of such risk forms, including the Finnish diabetes risk score (FINDRISC), are developed in Western populations, and the consensus is that risk scores should not be applied indiscriminately in different populations (i.e., low- and middle-income countries) [9].

The Saharawi people have been living in refugee camps around Tindouf, Algeria, since 1975. They live in a protracted environment in a harsh desert climate with a scarcity of fresh food and water. In addition to receiving monthly rations of basic commodities from international donors, food may be purchased in local shops. Sugar consumption is high while the level of physical activity is low [10] and a high prevalence of obesity, especially among Saharawi women, has been observed [11]. A study from 2019 with a convenient sample of adult Saharawi men and women attending health clinics found a prevalence of prediabetes of 10%, while more than 25% had undiagnosed T2D [12], underlining the need for a simple risk score with good predictive ability. Diabetes risk scores have been developed in Oman [13], Egypt [14], Iran [15], Kuwait [16] and MENA [17], but never in a protracted refugee setting. In the setting investigated, which had a poorly functioning health system, we believe that early intervention (before diabetes is diagnosed) is key to limiting complications and financial strains caused by the use of medication.

The aim of this study was to create a risk score for identifying subjects with undiagnosed prediabetes or T2D among Saharawi refugees in Algeria and compare the performance of this score to FINDRISC.

## Methods

### Aim, design and participants

A cross-sectional survey was carried out in 2014 in five refugee camps near Tindouf, Algeria. The total population in all five camps was estimated at approximately 165,000. Participants had to be 18 years of age to be eligible, while individuals who were sick, bedridden, unable to answer questions (for any reason), and women who were pregnant were excluded from the study. This study was part of a larger project aiming to assess metabolic risk factors in the Saharawi population by collecting data on dietary intake, obesity, stress, and physical activity. Sample size calculations were performed using Open Source Epidemiologic Statistics for Public Health (OpenEpi). The sample size (n = 360) was determined based on an estimated prevalence of an inadequate diet of 50% with a 5% significance level and an 80% confidence interval. Due to unequal numbers of inhabitants in the five refugee camps, a probability proportional to size (PPS) method, assuming a 50/50 gender balance, was used to select participants from each camp. A three-stage cluster sampling was performed. First a camp, then a household, and then one man and one woman from each household were selected. During the study period, 52 participants withdrew, due mainly to work obligations or personal or family illness, and two participants were excluded. Forty-nine additional participants were randomly recruited, so that the final sample size consisted of 355 participants, 175 men and 180 women.

### Data collection

Weight was measured in light clothing using digital Tara scales produced for UNICEF (SECA 890; SECA, Hamburg, Germany). Participants’ height, without shoes but with light headwear for women, was measured to the nearest 0.1 cm using an ultrasonic metre from Soehnle Professional or a UNICEF portable stadiometer if the participant had a similar or taller height than the field worker doing the measurements. Waist circumference was measured by trained personnel, midway between the ileac crest and lowest rib using an ergonomic measuring tape from SECA (SECA 201; SECA, Hamburg, Germany). BMI was calculated as body weight divided by body height squared (kg/m^2^). BMI cut-offs for being overweight and obese [18] and health risk based on waist circumference [19], as proposed by the WHO, were applied.

Cases were selected based on HbA1c levels. Blood samples were collected by the finger prick method, and HbA1c tests were performed using Quo-Test A1C test cassettes (EKF Diagnostics, Cardiff, England). Control testing of the equipment was performed weekly, plus for each new shipment of test cassettes, in case of implausible results or concern that the cassette had not been stored appropriately. The measuring device used was Afinion AS1000 (Abbott, Oslo, Norway). The resulting hbA1c values were transformed from percent to mmol/mol based on the following formula: HbA1cNGSP [%] = 0,09148 * HbA1cIFCC [mmol/mol] + 2,152 [20]. We used the American Diabetes Association (ADA) cut-offs to categorize participants as pre- or diabetic (HbA1c ≥ 39 mmol/mol )[21]. Risk scores were calculated based on anthropometric measurements and data on health status obtained from questionnaires.

The variables to include in the risk score were selected from previous publications where diabetes risk scores have been developed [13, 17, 22]. The variables included gender, age, BMI, waist circumference (WC), history of medication for high blood pressure, physical activity (PA), daily intake of fruits and vegetables, and diabetes in a first-degree relative. For level of physical activity, the International Physical Activity Questionnaire (IPAQ) was used. Participants were asked how many of the preceding seven days they had been physically active, and how many hours and minutes they had been physically active on each occasion. The same questions were repeated for activity of rigorous, moderate, and low intensity, and summed up for total time of physical activity (PA) Intake of fruits and vegetables was assessed by asking, “Do you eat vegetables or fruit every day?” where potatoes and fruit juice were excluded. To assess the final variables, participants were asked, “Have you ever taken medication for high blood pressure on a regular basis?” And finally, “Have any of your family members been diagnosed with type 1 or type 2 diabetes?”

There are a few differences between the FINDRISC variables and the variables tested in this study. Age was categorized into three groups (< 45, ≥ 45 and < 60 and ≥ 60 years) due to few participants in some groups, as compared to four groups in FINDRISC (where the age span 45–60 is categorized as two separate groups). Berries were excluded from the “How often do you eat fruits, vegetables, and berries” variable because consumption of berries in the camps is rare. Finally, we could not determine whether diabetes had been diagnosed in first- or second-degree relatives in our sample, so we only asked about first-degree relatives such as parent, sibling, or child, while FINDRISC also includes information on second-degree relatives.

### Statistics

Statistical analysis was performed using Statistical Package for the Social Sciences (SPSS) version 26 (SPSS Inc., Chicago, IL, USA). Participants who were already diagnosed with prediabetes or T2D (*n* = 22), were anaemic, or were using iron supplements or anti-psychotic medications (*n* = 24), potentially influencing HbA1c levels, were excluded from analysis. Continuous variables were presented as medians and minimum and maximum values since they were not normally distributed. Differences between groups were assessed by the Mann-Whitney U test for continuous variables and Pearson's Chi square test if variables were categorical. Associations between risk factors and pre-T2D and T2D were assessed by logistic regression. First, bivariate analysis was performed for each predictor and the outcome (HbA1c ≥ 39 mmol/mol; 5,7%). All variables were re-entered, and backwards multiple logistic regression with a significance level of 0.05 was used to arrive at the final multivariable model.

The final multivariable model was internally validated by bootstrapping using 1000 bootstrap samples to assess overfitting (i.e. better model performance in the development sample than in new samples with other subjects) [23], providing shrinkage factors for adjusting regression coefficients and adjusted model intercepts for use in prediction formulas, and to assess optimism-corrected model performance measures [24]. Internal validation was conducted using the R package rms [25] and Stata version 17 (StataCorp LLC, College Station, TX). Adjusted coefficients after shrinkage are presented. We assessed the model performance in terms of the R-squared (R^2^) for logistic regression, C-statistics (area under the receiver operating characteristic curve), calibration slope (where 1.00 is ideal and estimates below 1.00 indicate overfitting), and calibration-in-the-large (where 0 is ideal and estimates below 0 indicate that predicted probabilities are higher than the observed proportions). The model performance was estimated as apparent (estimated directly from a dataset that was used to develop the prediction model), training (average performance in each of the bootstrap samples with replacement), test (average estimate determined by the developing model in each bootstrap sample, and applying the bootstrap model in the original sample), optimism (the average difference between the model performance in bootstrap data and test performance in the original dataset), and optimism corrected (subtracting average optimism from apparent performance). The apparent and optimism-corrected estimates are reported. A calibration plot for the apparent performance of the Saharawi diabetes risk score was developed using PMCALPLOT in Stata [26].

The risk score was further calculated by adding a score for each regression coefficient, which showed a significant association with the outcome, using Schneeweiss’s scoring system. In this scoring system, weights are increased by 1 for every 0.3 increase in the β. Values below 0.15 get the weight 0 while values between 0.15 and 0.45 get the weight 1, 0.45-0.75 the weight 2, 0.75 and 1.05 the weight 3, 1.05 and 1.35 the weight 4, 1.35 and 1.65 the weight 5, 1.65 and 1.95 the weight 6 and finally 1.95 and 2.25 the weight 7 [27]. The sum of these scores was then analysed against the outcome in AUROC analysis to assess the performance of the simplified score. The Youden Index (the point on the ROC curve farthest from the diagonal line) [28] was used to determine the optimal cut-off. This cut-off and cross-tab analysis were finally used to calculate sensitivity, specificity, and positive and negative predictive values for the simplified risk score. AUROC and cross-tab analysis based on the optimal cut-off were also performed for FINDRISC with prediabetes or T2D as outcome. The risk score calculated from the corrected estimates performed virtually identically to the original risk score; thus, results based on the originally developed score are presented.

## Results

 Characteristics of the study population for non-cases and cases are presented in Table 1. The prevalence of prediabetes or T2D in the sample was 14%. Median (min, max) BMI was 23.3 (15.7, 42.7) among non-cases and 29.5 (18.1, 47.8) among cases (*p* < 0.001), while corresponding numbers for WC were 89.0 (60.9, 130.0) and 100.0 (80.0, 118.0) (*p* < 0.001) for women and 78.0 (62.5, 117.0) and 94 (57.6, 112.0; p 0.01) for men, respectively.

**Table 1:** Background variables (*n* = 308)

	Non-cases: (HbA1_**c**_ < 39 mmol/mol) (*n* = 266)	Cases (HbA1_**c**_ ≥ 39 mmol/mol) (*n* = 42)	***P***-value
**HbA1**_**c**_ (mmol/mol), median (min, max)	34.4 (23.5, 38.8)	42.1 (39.9, 118.6)	<0.001^a^
**Men**, n (%)	142 (90.4)	15 (9.6)	
**Women,** n (%)	124 (82.1)	27 (17.9)	0.03^b^
**Age**, median (min, max)	35 (18, 90)	52 (18, 76)	<0.001^a^
**Age groups**			
< 45 years, n (%)	174 (65.4)	13 (31.0)	
≥ 45 and < 60 years, n (%)	46 (17.3)	16 (38.1)	
≥ 60, n (%)	46 (17.3)	13 (31.0)	<0.001^b^
** Married**, n (%)	144 (54.1)	29 (69.0)	0.07^b^
**Educational level**			
None, n (%)	71 (26.7)	21 (50.0)	
< 6^th^ grade, n (%)	58 (21.8)	11 (26.2)	
7–9^th^ grade, n (%)	83 (31.2)	3 (7.1)	
10–12^th^ grade, n (%)	41 (15.2)	5 (11.9)	
Higher education	13 (4.9)	2 (4.8)	<0.01^b^
** Currently working**, n (%)	170 (63.9)	30 (71.4)	0.34^b^
** BMI**^**c**^, median (min, max)	23.3 (15.7, 42.7)	29.5 (18.1, 47.8)	<0.001^a^
**BMI categories**			
Lower than 25 kg/m^2^, n (%)	160 (63.5)	7 (17.1)	
25-30 kg/m^2^, n (%)	57 (21.4)	16 (39.0)	
Higher than 30 kg/m^2^, n (%)	40 (15.0)	18 (43.9)	<0.001^b^
**Waist circumference,** median (min, max)			
Women	89.0 (60.9, 130.0)	100.0 (80.0, 118.0)	<0.001
Men	78.0 (62.5, 117.0)	94 (57.6, 112.0)	0.01
**Waist circumference categories**^**d**^			
W: < 80 cm or M: < 94 cm, n (%)	158 (59.4)	6 (14.3)	
W: ≤ 88 cm or M: ≤ 102 cm, n (%)	40 (15.0)	8 (19.0)	
W: > 88 cm or M: > 102 cm, n (%)	68 (25.6)	28 (66.7)	<0.001^b^
**PA**^**e**^ ≥ **30 minutes per day**, n (%)	129 (48.7)	15 (37.5)	0.18^b^
**Daily consumption of FV**^**f**^, n (%)	91 (34.2)	13 (31.0)	0.70^b^
**Medication for high blood pressure**, n (%)	23 (8.6)	4 (9.5)	0.91^b^
**First degree relative**^**g**^**diagnosed with diabetes**, n (%)	44 (16.8)	11 (26.2)	0.14^b^

^a^ Mann-Whitney test for difference between medians

^b^ Chi square or Fischer exact test

^c^ BMI: *Body mass index*

^d^ Waist circumference categories proposed by WHO [19], W: W*omen*; M: M*en*

^e^ PA: *Physical activity at any level of intensity*

^f^ FV: *Fruits and vegetables*

^g^ Parent, sibling, or child

Associations between risk factors and undiagnosed prediabetes or T2D are presented in Table 2**.** Variables significantly associated with increased risk of prediabetes or T2D in bivariate models were female gender, increasing age, higher BMI, and higher WC, while physical activity, intake of fruits and vegetables, use of blood pressure medication, and diabetes in first-degree relative were not significantly associated with the outcome (HbA1c ≥ 39 mmol/mol).

**Table 2:** Associations between diabetes risk factors and undiagnosed pre-or type 2 diabetes (HbA1_c_ ≥ 39 mmol/mol), Saharawi refugee camps Algeria, 2014; from logisitic regressions (*n* = 301 in adjusted analysis)

	Bivariate model	Adjusted model^**a**^	Regression coefficient (B)	Score^**b**^	Regression coefficient after shrinkage (B)^**c**^	Score^**b**^
	OR (95% CI)	OR (95% CI)				
Intercept			-4.16		-3.76	
**Gender** (female as reference)	0.49 (0.25, 0.95)					
**Age** (vs.< 45 years)						
≥ 45 and < 60 years	4.66 (2.09, 10.37)	3.62 (1.51, 8.68)	1.29	4	1.10	4
≥ 60	3.78 (1.64, 8.72)	3.60 (1.45, 8.91)	1.28	4	1.10	4
**BMI**^**c**^ (vs. < 25 kg/m^2)^						
≥ 25 and < 30 kg/m^2^	6.78 (2.65, 17.31)	4.08 (1.40, 11.85)	1.41	5	1.21	4
≥ 30 kg/m^2^	10.86 (4.25, 27.73)	3.74 (1.12, 12.55)	1.32	4	1.13	4
**WC**^**d**^ (vs. F: < 80 cm/M: < 94 cm)						
F: ≥ 80 and < 88 cm/M: ≥ 94 and < 102 cm	5.27 (1.73, 16.04)	2.31 (0.65, 8.12)	0.84	3	0.72	2
F: ≥ 88/M: ≥ 102	10.84 (4.29, 27.38)	3.57 (1.08, 11.77)	1.27	4	1.09	4
**Physical activity** (vs. none)						
At least 30 min per day	0.63 (0.32, 1.25)					
**Intake of fruits and vegetables** (vs. no)						
At least once per day	0.83 (0.41, 1.66)					
**Blood pressure medication** (vs. no)	0.99 (0.97, 1.02)					
**Diabetes in first**-**degree relative** (vs. no)	1.76 (0.82, 3.76)					

^a^ Adjusted model created by backward elimination (starting with all variables tested in bivariate models), variables significantly associated with undiagnosed prediabetes or T2D were kept in the model.

^b^ Based on Schneeweiss’s scoring system [27] (Schneeweiss, 2003 #258)

^c^ Calculated from internal validation using Bootstrap analysis

^d^ Body mass index

^e^ Waist circumference

The optimism corrected R^2^ after Bootstrap analysis was 0.23 (compared to 0.28 in the original model), the calibration slope was 0.86, calibration-in-the-large was -0.17, and the C-statistics was 0.80 (compared to 0.82 in the original model).

**Fig. 1:**
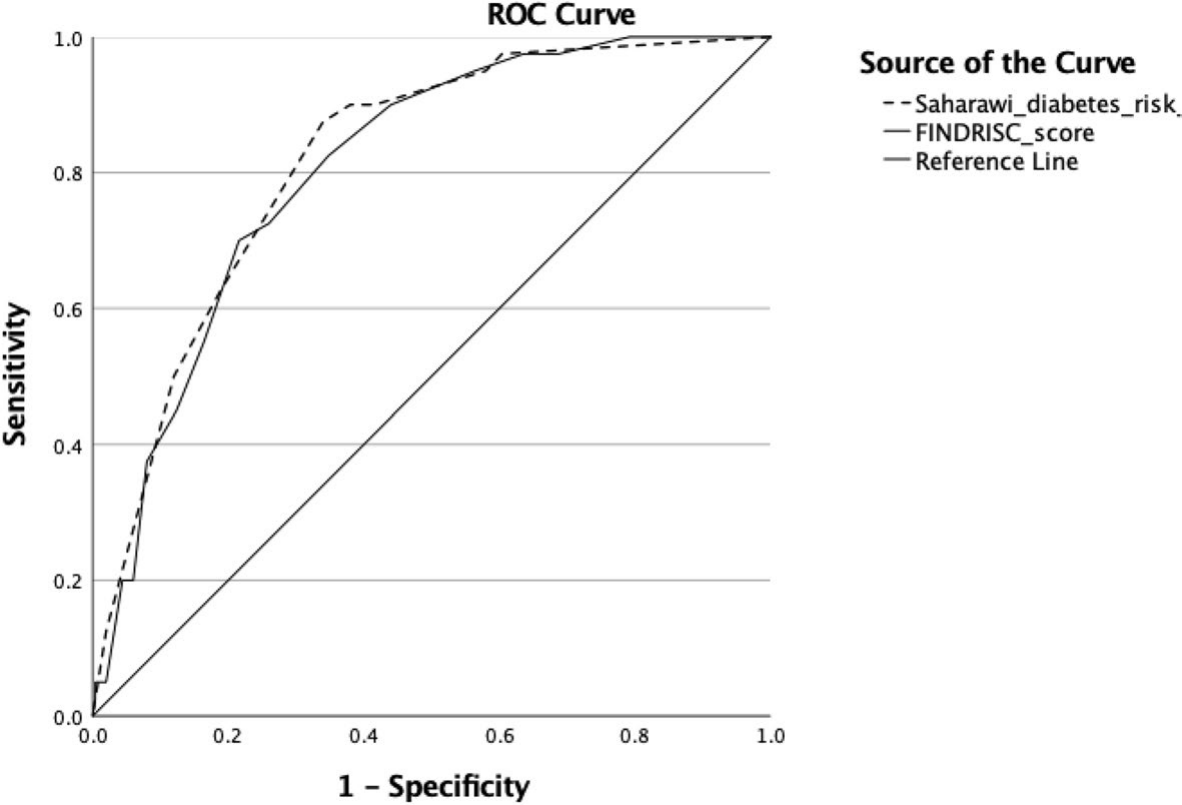
Area Under the ROC curve. Comparison of Area Under the ROC curve (AUROC) for the Saharawi diabetes risk score and FINDRISC

**Fig. 2:**
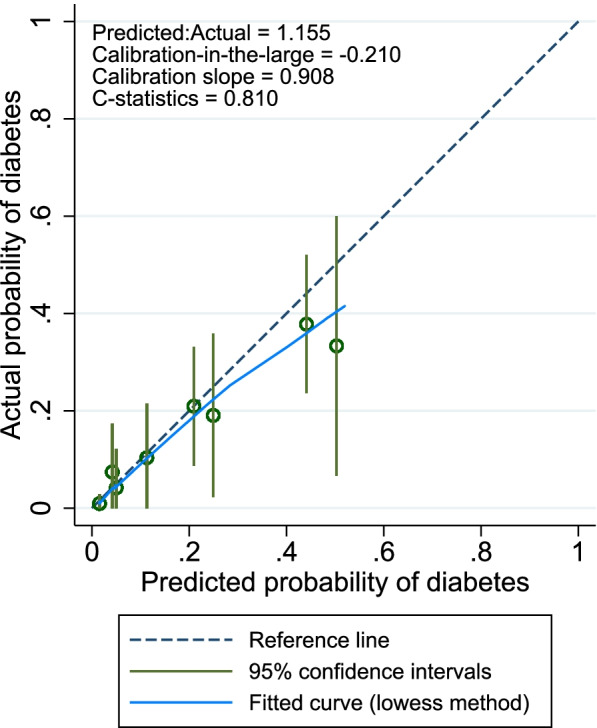
Calibration plot of prediction model performance. Calibration plot of prediction model performance using a random selected bootstrapped sample with the 8 different percentiles of predicted probabilities. The reference line indicates a perfect relationship between predicted and actual probabilities and the fitted curve using the lowess method shows the observed relationship. C-statistics is the area under the receiver operating characteristics curve

The diagnostic accuracy of the Saharawi diabetes risk score compared to the FINDRISC score is presented in Table 3. Sensitivity, reflecting true positive cases, was 87.8% with the Saharawi diabetes risk score and 72.5% with FINDRISC. Corresponding numbers for specificity (true negative cases) were 65 and 74%. The AUROC (C.I) was 0.817 (0.756, 0.879) and 0.808 (0.745, 0.870) for the Saharawi diabetes risk score and FINDRISC, respectively (Fig. 1). The calibration plot of prediction model performance using a random selected bootstrapped sample is presented in Fig. 2.

**Table 3:** Comparison of performance of the Saharawi diabetes risk score and FINDRISC (*n* = 307)

	Saharawi diabetes risk score	FINDRISC
Area under the ROC curve, AUC (C.I)	0.817 (0.756, 0.879)	0.808 (0.745, 0.870)
Empirical cut-off	8	9
Sensitivity, %	87.8	72.5
Specificity, %	65.0	74.0
Positive predictive value, %	27.9	30.9
Negative predictive value, %	97.2	94.4

The distribution of cases and non-cases at different levels of the Saharawi diabetes risk score is presented in Table 4**.** The sum of ranks was significantly different between cases and non-cases for both women and men (*p* < 0.001). Most cases, both among women (96%) and men (80%), had a Saharawi diabetes risk score above 7 (out of a maximum score of 13).

**Table 4:** Distribution of cases and non-cases for women and men at different levels of the Saharawi diabetes risk score (*n* = 308)

Saharawi diabetes risk score	Cases women *N* = 26	Non-cases women *N* = 124	Cases men *N* = 15	Non-cases men *N* = 142
0-2, n (%)	0 (5)	31 (25.0)	1 (6.7)	74 (52.1)
3-6, n (%)	1 (3.8)	19 (15.3)	2 (13.3)	39 (27.5)
7-10, n (%)	10 (38.5)	52 (41.9)	6 (40.0)	18 (12.7)

## Discussion

In resource-constrained settings, a simplified risk score for prediabetes or T2D may be a useful screening tool to decide who should receive lifestyle advice to prevent the progression of the disease and who should be referred to blood sample analysis. Preferably, the tool should be so simple that it can be applied by the general population [29] and should not include any lab analysis [8, 13]. Previous studies indicate that the performance of risk scores may be low when there is heterogeneity of characteristics between settings [30]. The Saharawi refugee camps provide a setting unlike most others, where the population has limited opportunities for PA [10] and is dependent on food aid. A risk score adapted to this population is therefore an important contribution to improving public health in the population. The simplified Saharawi diabetes risk score developed in this study, including only age, BMI, and WC, showed good predictive ability with particularly high sensitivity and high negative predictive value, both slightly higher than FINDRISC. Meanwhile, the outcome in our study included prediabetes which differs from the outcome for which FINDRISC was originally developed. This likely had an influence on the performance of FINDRISC.

The risk factors most strongly associated with diabetes in our study were age, BMI, and WC, which are commonly found to be associated with risk of diabetes in various settings [2, 17, 22]. Fewer risk factors were included in our multivariable model compared to numerous other risk scores developed [2, 13, 15], possibly due to too few cases and moderate statistical power in our study. The model also showed some overfitting after internal validation with a mean calibration slope of 0.86 and calibration-in-the-large of -0.17. This is likely due to moderate statistical power compared to the number of variables investigated. Interestingly, the odds of diabetes did not seem to increase from the middle to the oldest age group (> 60 years). This contradicts findings from studies in high-income countries [22] and Oman [13] but corresponds to results from the DETECT-2 project, where it was found that in India and Africa, the odds of diabetes were lower in those > 59 than in younger age groups [31]. At the same time, the group with a BMI 25–30 had nearly similar odds of diabetes in the adjusted model as those with a BMI > 30. This might be due to an interaction with age in which older individuals may be less prone to a Westernized diet and subsequent risk of becoming overweight or obese. On the other hand, dietary intake and physical activity level appeared not to impact the risk of diabetes, and it has been proposed that the performance of diabetes risk scores would be equally sound if questions about diet and physical activity were excluded [32]. To this end, a systematic review found that the performance of various scores to detect undiagnosed T2D did not differ by the number of variables included [33].

The AUC for the Saharawi diabetes risk score was similar to [15, 16] or slightly higher [13] than risk scores developed in other MENA countries. A meta-analysis comparing the performance of diabetes risk scores found that self-developed risk scores usually perform better than the validation of existing risk scores in new populations [2]. Consequently, the AUC for FINDRISC in our population was also higher than what has been found in Saudi Arabia, the United Arab Emirates [17], and Algeria [17, 34]. The relatively high AUC found in this study, both for the Saharawi diabetes risk score and FINDRISC, may indicate that the Saharawi population has a clear risk profile with regard to prediabetes or T2D. Life in the refugee camps offers less variation in living conditions (i.e. food intake, PA) than most other settings. Apart from risk factors based on anthropometry, there were few significant differences between cases and non-cases, which might explain the similar performance of the simplified risk score and FINDRISC. On the one hand, this might be due to moderate statistical power. On the other hand, fieldworkers did not have access to medical records, and all other variables were based on self-report increasing the risk of recall bias. Applying the European cut-offs for BMI and WC could potentially have weakened associations since, for instance, WC cut-offs for health risk among men [19], and BMI cut-offs for both genders [34] are known to differ between population groups. It is thus probable that applying cut-offs validated for the group studied could improve the predictive power of risk scores, including ours, even further.

Sensitivity for the Sahrawi diabetes risk score was high and slightly higher than for FINDRISC. The minimization of missing cases is desirable, especially when the screening test is simple and inexpensive [34] as in our study. Further, the negative predictive value was very high. This indicates that the test performs fine in excluding those without a need for further blood tests, which is a great advantage when resources are limited. At the same time, the positive predictive value was low, which has also been seen in other studies [13]. This is commonly found when the prevalence of the outcome is low, which might have been the case here. The low prevalence of pre-diabetes despite a high prevalence of overweight and obesity in this study merits attention, and recent reports seem to suggest that the use of HbA1c may underestimate diabetes risk in non-Western population groups [35, 36]. Finally, in a systematic review of the performance of 31 diabetes risk forms, FINDRISC had the highest combination of sensitivity (81%) and specificity (76%) [33], comparable to what was found both for the Saharawi diabetes risk score and FINDRISC in this study.

The main strengths of the study are a randomly selected sample covering a large age span, the fact that participants were interviewed, a high response rate, and inclusion of Saharawi men, since most previous health surveys in the camps have focused on women and children [11]. The main weakness is the moderate sample size with few cases included, which may lead to unstable effect estimates as indicated by signs of overfitting after internal validation with bootstrapping. An overfitted statistical model may limit its generalizability outside the original dataset The Saharawi refugees, for the most part, live under reasonably similar conditions, and although the sample likely is representative of the population investigated, the findings probably have limited generalizability outside of the camps. Further, the data were cross-sectional in nature, identifying prevalent cases with prediabetes or T2D instead of incident cases or individuals at risk, which is the main purpose of a risk score. However, it is unlikely that risk factors for prediction of prevalent and incident cases differ to any significant degree [29]. In addition to iron deficiency, HbA1_c_ may be influenced by both genetic and medical conditions for which this study did not provide any data. Finally, categorization of risk factors included in the risk score simplifies it’s use [8], but neglects the great variation in risk within the different categories of anthropometry, and so slightly different association could have been found if continuous variables had been used.

In conclusion, we found that a simplified risk score consisting of age, BMI, and WC performed well to identify prevalent prediabetes or T2D among Saharawi refugees. The Saharawi diabetes risk score performed slightly better than FINDRISC, and based on simplicity, should be the preferred choice for early diabetes screening. Meanwhile, the inclusion of anthropometric measures requiring stadiometer, scale, and measuring tape makes its use more feasible for health care workers than for the general population. Although the score performed similarly to scores developed in other MENA settings, its discriminatory ability should ideally be validated in new larger datasets from the Saharawi population.

## Data Availability

The datasets used and/or analysed during the current study are available from the corresponding author on reasonable request.

## Abbreviations

T2D: Type 2 diabetes
ADA: American Diabetes Association
AUROC: Area Under the Receiver Operating Curve
AUC: Area Under the Curve
BMI: Body mass index
IDF: International Diabetes Federation
WC: Waist circumference
IPAQ: International Physical Activity Questionnaire
SPSS: Statistical package for the social sciences

## References

[1] NCD Risk factor collaboration (2016). Worldwide trends in diabetes since 1980: a pooled analysis of 751 population-based studies with 4.4 million participants. Lancet..

[2] Collins GS, Mallett S, Omar O, Yu LM. Developing risk prediction models for type 2 diabetes: A systematic review of methodology and reporting. BMC Med. 2011;9:1–14 10.1186/1741-7015-9-103 PMC3180398 21902820

[3] Diabetes in MENA. https://idf.org/our-network/regions-members/middle-east-and-north-africa/diabetes-in-mena.html. Accessed 11 Nov 2021.

[4] NCD Risk Factor Collaboration - Africa group. Trends in obesity and diabetes across Africa from 1980 to 2014: an analysis of pooled population-based estimates. Int J Epidemiol. 2017;46(5):1421–32. 10.1093/ije/dyx078 PMC5837192 28582528

[5] Beagley J, Guariguata L, Weil C, Motala AA (2014). Global estimates of undiagnosed diabetes in adults. Diabetes Res Clin Pract..

[6] Selph S, Dana T, Blazina I, Bougatsos C, Patel H, Chou R. Screening for type 2 diabetes mellitus: A systematic review for the U.S. preventive services task force. Ann Intern Med. 2015;162(11):765–76. 10.7326/M14-2221 25867111

[7] International Diabetes Federation Guideline Development Group (2014). Global guideline for type 2 diabetes. Diabetes Res Clin Pract..

[8] Bernabe-Ortiz A, Perel P, Miranda JJ, Smeeth L (2018). Diagnostic accuracy of the Finnish Diabetes Risk Score (FINDRISC) for undiagnosed T2DM in Peruvian population. Prim Care Diabetes..

[9] Schwarz PE, Li J, Lindstrom J, Tuomilehto J (2009). Tools for predicting the risk of type 2 diabetes in daily practice. Horm Metab Res..

[10] Andersen E, Kjellså I, Hjellset VT, Henjum S. Insufficient physical activity level among Sahrawi adults living in a protracted refugee setting. BMC Public Health. 2021;21(166) 10.1186/s12889-021-10217-w PMC7816400 33468100

[11] Henjum S, Barikmo I, Strand TA, Oshaug A, Torheim LE (2012). Iodine-induced goitre and high prevalence of anaemia among Saharawi refugee women. Public Health Nutr..

[12] Carretero-Anibarro E, Hamud-Uedha M (2019). Prevalence of undiagnosed type 2 diabetes mellitus in the sahrawi population of the sahrawi refugee camps of Tindouf. Algeria. Med Clin (Barc)..

[13] Al-Lawati JA, Tuomilehto J (2007). Diabetes risk score in Oman: A tool to identify prevalent type 2 diabetes among Arabs of the Middle East. Diabetes Res Clin Pract..

[14] Tabaei BP, Herman WH (2002). A multivariate logistic regression equation to screen for diabetes: Development and validation. Diabetes Care..

[15] Bozorgmanesh M, Hadaegh F, Ghaffari S, Harati H, Azizi F (2011). A simple risk score effectively predicted type 2 diabetes in Iranian adult population: Population-based cohort study. Eur J Public Health..

[16] Al Khalaf MM, Eid MM, Najjar HA, Alhajry KM, Doi SA, Thalib L. Screening for diabetes in kuwait and evaluation of risk scores. East Mediterr Health J. 2010. 20799528

[17] Handlos LN, Witte DR, Almdal TP, Nielsen LB, Badawi SE, Sheikh ARA (2013). Risk scores for diabetes and impaired glycaemia in the Middle East and North Africa. Diabetic Med..

[18] World Health Organization. BMI classification. http://apps.who.int/bmi/index.jsp?introPage=intro_3.html. Accessed 1 Jan 2020.

[19] World Health Organization (2008). Waist circumference and Waist-hip ratio - Report of a WHO Expert Consultation.

[20] Norwegian Organization for Quality Improvement of Laboratory Examinations. https://www.noklus.no/Kursogveiledning/HbA1c_omregning.aspx. Accessed 05 Nov 2019.

[21] American Diabetes Association (2018). Classification and Diagnosis of Diabetes: Standards of Medical Care in Diabetes - 2018. Diabetes Care..

[22] Lindstrom J, Tuomilehto J (2003). The diabetes risk score: a practical tool to predict type 2 diabetes risk. Diabetes Care..

[23] Steyerberg E (2019). Clinical prediction models: a practical approach to development, validation, and updating.

[24] Steyerberg EW, Bleeker SE, Moll HA, Grobbee DE, Moons KGM (2003). Internal and external validation of predictive models: a simulation study of bias and precision in small samples. J Clin Epidemiol..

[25] R Core Team. A language and environment for statistical computing. 2020. https://www.r-project.org/.

[26] Ensor J, Snell KIE, Martin EC (2018). PMCALPLOT: Stata module to produce calibration plot of prediction model performance.

[27] Schneeweiss S, Wang PS, Avorn J, Glynn RJ (2003). Improved comorbidity adjustment for predicting mortality in Medicare populations. Health Serv Res..

[28] Šimundić A-M (2009). Measures of Diagnostic Accuracy: Basic Definitions. EJIFCC..

[29] Herman WH (2009). Predicting risk for diabetes: choosing (or building) the right model. Ann Intern Med..

[30] Rathmann W, Martin S, Haastert B, Icks A, Holle R, Löwel H (2005). Performance of screening questionnaires and risk scores for undiagnosed diabetes: The KORA survey 2000. Arch Internal Med..

[31] Glumer C, Vistisen D, Borch-Johnsen K, Colagiuri S, Collaboration D-. (2006). Risk scores for type 2 diabetes can be applied in some populations but not all. Diabetes Care..

[32] Bergmann A, Li J, Wang L, Schulze J, Bornstein SR, Schwarz PE (2007). A simplified Finnish diabetes risk score to predict type 2 diabetes risk and disease evolution in a German population. Horm Metab Res..

[33] Brown N, Critchley J, Bogowicz P, Mayige M, Unwin N (2012). Risk scores based on self-reported or available clinical data to detect undiagnosed type 2 diabetes: a systematic review. Diabetes Res Clin Pract..

[34] Azzouz A, Guerchani M-K, Lyes Y, Hannachi R, Bahous H, Meftah A, Mimouni SMB (2014). Apport du score de risque finlandais FINDRISC dans l’identification de la dysglycémie dans une population algérioise, Algérie. Médecines des maladies Métaboliques..

[35] Hjellset VT, Bjorge B, Eriksen HRHA (2011). Risk factors for type 2 diabetes among female Pakistani immigrants: the InvaDiab-DEPLAN study on Pakistani immigrant women living in Oslo, Norway. J Immigr Minor Health..

[36] Araneta MRG, Kanaya AM, Hsu WC, Chang HK, Grandinetti A, Boyko EJ (2015). Optimum BMI cut points to screen Asian Americans for type 2 diabetes. Diabetes Care..

